# Editorial: Decoding biofilm resilience: integrative multi-omics approaches and novel disruption strategies

**DOI:** 10.3389/fcimb.2025.1756728

**Published:** 2026-01-15

**Authors:** Sampathkumar Ranganathan, Hemavathy Nagarajan, Siddhardha Busi, Palanichamy Esakkiraj, Paramanantham Parasuraman, Ramya Chandar Charles Mariasoosai, Umashankar Vetrivel

**Affiliations:** 1 Centre for Bioinformatics, Kamalnayan Bajaj Institute for Research in Vision and Ophthalmology, Vision Research Foundation, Sankara Nethralaya, Chennai, Tamil Nadu, India; 2 Department of Chemical Engineering, Konkuk University, Seoul, Republic of Korea; 3 Department of Microbiology, School of Life Sciences, Pondicherry University, Puducherry, India; 4 Center for Global Health Research, Saveetha Medical College, Chennai, India; 5 Department of Chemistry and Biochemistry, University of Texas at Dallas, Austin, TX, United States; 6 Department of Virology and Biotechnology/Bioinformatics Division, Indian Council of Medical Research-National Institute for Research in Tuberculosis, (ICMR-NIRT), Chennai, India; 7 Academy of Scientific and Innovative Research (AcSIR), Ghaziabad, Uttar Pradesh, India

**Keywords:** antimicrobial resistance, antivirulence, biofilm, multiomics, quorum Sensing (QS)

Biofilms represent one of the most resilient microbial defense mechanisms, contributing substantially to chronic infection, antimicrobial resistance (AMR), and treatment failure across clinical and environmental settings (Cruz et al., 2021). Although classical microbiology has brought into focus significant virulence characteristics, multi-omics technologies that combine genomics, transcriptomics, proteomics, metabolomics, lipidomics, and the latest imaging methods have revolutionized the way we study biofilms, both on a single-cell and community basis. These studies indicate that biofilms are complex entities that demonstrate intricate regulatory loops, metabolic adaptability, and stress-adaptation pathways, rendering them challenging to eliminate. Multi-omics analysis in recent studies suggests that biofilm formation is a dynamic process involving multiple stages and influenced by genetic factors: control, metabolic adaptation, and environmental signatures. Significant features include adhesion factors and the genes involved in the biosynthesis of extracellular polymeric substance (EPS) and quorum-sensing (QS) components. The QS networks are efficient in managing group behaviors as well as metabolic reprogramming in response to oxygen and nutrients (Rather et al., 2021). The gradients lead to the development of persister cells and the upregulation of efflux pump function, thereby enhancing antibiotic resistance (Alagesan et al., 2025; Arumugam et al., 2024; Papenfort and Bassler, 2016).

This Research Topic brings complementary studies that collectively represent how multi-omics, quorum sensing (QS) analysis, and mechanistic investigation of antibiotic compounds are transforming our understanding of biofilm persistence and vulnerability in biofilm research.

## Biofilm formation and antimicrobial resistance in *Klebsiella pneumoniae*

Li et al. offer an exhaustive and mechanistically based synthesis of the molecular interactions and cooperation between biofilm development and antibiotic resistance in the high-priority, ESKAPE pathogen *Klebsiella pneumoniae*. The authors have utilized genomic, transcriptomic, and phenotypic evidence to demonstrate that biofilm formation is not a single virulence phenotype, but rather a concerted effort involving the production of capsular polysaccharide, fimbrial adhesion systems, quorum sensing (QS), and multidrug efflux activity. More importantly, the review has established a strong correlation between biofilm positivity and multidrug-resistant (MDR) and carbapenem-resistant phenotypes, indicating that biofilms are the primary contributors to persistence, therapeutic failure, and chronicity in *K. pneumoniae* infections. Li et al. highlight biofilm disruption as a precondition for the reestablishment of antibiotic activity and resistance to antimicrobial selective pressures in this clinically significant pathogen by converging on the emergence of biofilm-related strategies. *K. pneumoniae* biofilm formation is a process that is tightly controlled and multiphase, which forms the basis of protracted existence in adverse host and hospital environments (Balestrino et al., 2008; Boddicker et al., 2006). Type 3 fimbriae (*mrk*ABCDF) are under c-di-GMP signaling and controlled by the transcriptional regulator MrkH. Structural integrity and immune evasion are mediated by capsular polysaccharides and lipopolysaccharides (e.g., wza, wzc, wcaG) (Wu et al., 2011), and surface attachment and biofilm formation are mediated by type 3 fimbriae (*mrk*ABCDF) (Wilksch et al., 2011). Such mechanisms are also mediated by the LuxS/AI-2 quorum-sensing system and supported by multidrug efflux systems (Balestrino et al., 2008; Chen et al., 2020). They collectively promote the synthesis of an extracellular polymeric substrate (EPS) with high polysaccharide, protein, and extracellular DNA content, which provides a protective niche that promotes adhesion to medical devices and host tissues, and protects against immune clearance of bacterial communities. The biofilm regime significantly increases the antimicrobial tolerance and resistance by three synergistic processes. First, the EPS matrix limits the penetration of antibiotics, thereby decreasing the concentration of effective drugs on the bacterial surface. Second, biofilm physiological heterogeneity gives rise to slow-growing and persister subpopulations, which are inherently more resistant to antibiotics. Third, biofilms maximize gene adaptation through the growth of efflux pumps and horizontal gene transfer. Although MDR, XDR, and carbapenem-resistant isolates usually possess strong biofilm phenotypes, the relationship is not univocal, with some highly resistant lineages having weak biofilm phenotypes as a phenomenon of evolutionary trade-offs between resistance determinants and biofilm fitness (Ashwath et al., 2022; Khodadadian et al., 2018; Vuotto et al., 2017). Because traditional antibiotics have only a limited ability to access cells within biofilms, the review demonstrates that the general strategy of approach has shifted towards treatment approaches that enhance the biofilm condition. The potentially useful ones include rational combinations of antibiotics (Bisso Ndezo et al., 2021; Moshynets et al., 2023; Ribeiro et al., 2023; Ribera et al., 2019; She et al., 2022), which result in greater penetration and resistance prevention, anti-membrane antimicrobial peptides (Artini et al., 2023; Cebrián et al., 2021; Chatupheeraphat et al., 2023; Fleeman and Davies, 2022; Kim et al., 2016), and the use of nanoparticles as a delivery platform (Sánchez-López et al., 2020; Sharma et al., 2019), which can promote intracellular drug delivery and ROS-mediated cell damage. At the same time, natural products and microbial metabolites that interfere with quorum sensing, EPS biosynthesis, and efflux activity, as well as bacteriophage-based therapies and capsule depolymerases that can break down biofilm matrices, offer highly specific solutions. Together, the strategies redefine biofilms as dynamic, resistance-promoting ecosystems, and biofilm-based intervention is a valuable avenue to achieve victory in recalcitrant *K. pneumoniae* infections and to suppress the growing menace of developing antimicrobial resistance.

## AI-2/LuxS quorum sensing in *Streptococcus equi subsp. zooepidemicus*

In the second study, Xie et al. include an extensive analysis of AI-2/LuxS-mediated quorum sensing (QS) in *Streptococcus equi subsp. zooepidemicus* (SEZ), which is a growing clinically significant zoonotic pathogen (Timoney, 2004). The authors employ a hybrid method, comprising the specific deletion of genes and global transcriptomics, as well as *in vivo* infection models, to demonstrate that LuxS serves as a crossroads linking the biofilm architecture, stress response, and host-pathogen relationships. It is essential to note that LuxS deficiency does not result in a drastic shift in mortality; instead, it alters the balance between growth and host reactions, indicating that QS is not a binary virulence switch. This study provides the first direct indication that the AI-2/LuxS signal regulates biofilm formation and pathogenic capability in SEZ, where QS interference may become an attractive strategy for anti-virulence (Ramić et al., 2023). SEZ has a preserved AI-2/LuxS QS system, since AI-2 production, which was dependent on LuxS, was detected. Remarkably, there are no remnants of deletion of luxS on the basal growth of bacteria, so LuxS appears to be a regulatory, but not a metabolic, fitness activity. This observation makes LuxS a universal signaling center that organizes population-level responses and physiological plasticity, but does not directly stimulate proliferation. In contrast to classical QS models that favor biofilm formation, LuxS in SEZ acts as a negative regulator of biofilm formation. It has been confirmed that the loss of luxS results in an increase in biofilm biomass, density, and structural complexity, as measured by crystal violet staining, scanning electron microscopy, and confocal laser scanning microscopy. The specificity of this effect is confirmed by restoration of the wild-type phenotype on complementation. These findings suggest that AI-2/LuxS signaling serves as a biofilm homeostasis signal, inhibiting unnecessary or energetically expensive biofilm growth without inducing surface colonization. Mechanistically, the Biofilm control LuxS-dependent is independent of traditional virulence-related surface characteristics. The deletion of luxS has no impact on the production of hyaluronic acid capsules, the cell surface hydrophobicity, or the hemolytic and adhesion to the epithelium. This separation of biofilm control by canonical adhesin and capsule determinants suggests that AI-2/LuxS signaling can regulate biofilm structure through downstream metabolic, stress-response, or regulatory signals, rather than directly controlling surface virulence factors. In addition to controlling biofilm, AI-2/LuxS signaling plays a crucial role in determining host immune responses. LuxS mutant ΔluxS triggers increased macrophage apoptosis and an enhanced production of pro-inflammatory cytokines (TNF-α, IL-1β, IL-6, and IL-18) induced by NF-κB, suggesting that LuxS suppresses the overproduction of pro-inflammatory cytokines within the body. LuxS deletion does not cause mortality in the host; however, it leads to excessive bacterial loads and severe inflammatory damage to the spleen *in vivo*.

These findings demonstrate that LuxS can be a crucial regulator of immune homeostasis and tissue-specific distribution, particularly in cases of acute mortality. Transcriptomic studies have shown that LuxS plays roles in large regulatory networks that control carbohydrate metabolism, transport, molecular chaperones, and heat-shock proteins, as well as silencing genes that regulate DNA replication and cell wall biosynthesis. Interestingly, CRISPR-Cas systems and stress resistance in the ΔluxS mutant increase, suggesting a limit on persistence-oriented phenotypes by LuxS (Nie et al., 2022; Waller and Robinson, 2013). This interaction between quorum sensing and genome defense, stress resilience, and long-term survival mechanisms establishes LuxS as the master controller of adaptive fitness. Overall, AI-2/LuxS quorum sensing within SEZ is part of the fine-tuning regulatory mechanism for biofilm formation, metabolic conditions, stress resistance, and host immune interactions. LuxS does not cause hyper-biofilm formation or hyper-inflammation, but rather inhibits them; in the event of infection, this would ensure as high a persistence and tissue adaptation as possible. The design of anti-virulence therapeutics is significantly implicated in this re-evaluation of QS as a homeostatic regulator.

## Parallels with the integrated quorum sensing system in *Pseudomonas aeruginosa*

Similar principles of regulation are found in *Pseudomonas aeruginosa*, where the Integrated Quorum Sensing (IQS) system complements the canonical Las–Rhl–Pqs hierarchy to organize virulence and biofilm development in response to environmental stress (Lee and Zhang, 2015; Raya et al.). Multi-omics studies suggest that IQS-mediated control can be induced by phosphate deficiency and metabolic stress, even in the absence of their upstream regulators, such as Las, which is commonly found in clinical isolates (Papenfort and Bassler, 2016). This rewiring of adaptive response underscores the ability of QS networks to enable *P. aeruginosa* biofilms to survive in the unfavorable conditions of the host and chronic infections. Notably, these systems-level understandings are directly therapeutic. Small-molecule QS inhibitors, such as the IQS-related compound B-11, repress the expression of rhlA and pqsA, and decrease the production of pyocyanin and rhamnolipids under phosphate-limited conditions, demonstrating that it is possible to regulate stress-responsive QS circuits, but not bacterial viability (Ochsner and Reiser, 1995; Pearson et al., 1994; Pesci et al., 1999). As resistance to antimicrobials is also escalating worldwide, these anti-QS methods offer a resistance-controlling alternative to traditional antibiotics. By combining results from models of LuxS-mediated biofilm restraint in SEZ and IQS-controlled virulence plasticity in *P. aeruginosa*, it becomes apparent that there is a common thread: biofilm behavior is regulated by adaptive stress-responsive regulatory networks, not merely by the activation of virulence. Cracking the code of such communication systems reveals exploitable weaknesses in biofilm-related pathogens. Targeting QS circuits, CRISPR-associated persistence mechanisms, and stress-adaptation responses is a promising approach to next-generation anti-biofilm and anti-virulence therapies, which complement antibiotics and reduce the selection pressure for the development of resistance.

## Mechanistic dissection of antibiofilm compounds JG-1 and M4

The final study gives a thorough mechanistic degradation of the antibiofilm molecules JG-1 and M4 in various clinically significant bacterial pathogens (Bennett et al.). Combining RNS sequencing, thermal proteome profiling, and targeted mutagenesis, the authors demonstrate that these compounds do not function through nonspecific degradation of the matrix or direct bactericidal action. Rather, JG-1 and M4 perturb protein interaction networks of core proteins that maintain biofilm integrity, demonstrating that biofilms are actively maintained and regulated-controlled networks (Huggins et al., 2018; Sandala et al., 2020). Bennett et al. present a framework for developing antibiofilm drugs at the systems level. JG-1 and M4 are highly active against antibiofilm without influencing the growth of planktonic bacteria, which confirms that bactericidal or bacteriostatic activities do not affect the potency of the two compounds. The two compounds are effective anti-browning agents that prevent biofilm formation and destabilize an established biofilm at sub-inhibitory levels. This antivirulence mechanism of action prevents the selective pressure of evolution of resistance, emphasizing a paradigm of therapy focused on pathological behavior rather than bacterial viability.

The ultrastructure of the biofilm has been investigated at a high level of resolution, and the study has shown that treatment with JG-1 or M4 results in a substantially different biofilm ultrastructure, characterized by reduced microcolony formation, decreased surface coverage, and a fragmented, loosely organized structure. There is also a loss of extracellular polymeric substances associated with these structural flaws, meaning that biofilm maturation and matrix stability are disrupted, rather than the initial adherence to the surface. The observation leads to the conclusion that biofilm integrity is a dynamically regulated state that can be disrupted. Both compounds inhibit quorum-sensing-associated pathways that regulate collective biofilm behaviors at the molecular level (Das et al., 2019).

Transcriptomic profiling reveals widespread downregulated genes involved in signal transduction, intercellular communication, and population density-dependent regulation. These effects indicate that JG-1 and M4 are effective quorum-sensing antagonists, which disrupt cell-cell communication between biofilm developmental programs and consequently break down coordinated community behavior. In addition to marking the presence of interference, JG-1 and M4 result in widespread metabolic and stress-response reprogramming. Critical pathways of carbohydrate metabolism, energy metabolism, and stress response are redesigned, changing the bacterial physiology toward a more unstable and dispersal-prone state, as opposed to the cooperative, stress-tolerant biofilm state. Notably, this reprogramming does not induce lethal stress responses, highlighting their activities as biofilm-disruptive but not cytotoxic agents. Although JG-1 and M4 overlap in their effects on antibiofilm production, the two compounds exhibit mechanistic biases: JG-1 is more specific to signaling and regulatory pathways, whereas M4 has a greater impact on metabolic pathways and processes mediated by the EPS. Such complementary approaches highlight their potential application as complements. More broadly, the research will provide a paradigm shift in the treatment of biofilms, not based on growth inhibition, but on the disruption of regulatory circuits that actively maintain biofilm resistance, which is a rational step in the development of long-term antibiofilm therapy that reduces resistance.

Overall, the research contributions to this topic paved the way for a more mechanistic understanding of biofilm resilience and the development of new treatment regimens, further advancing the domain in the context of clinical applications. Biofilms are the epicenter of chronic and recurrent infection and remain a significant global threat in AMR. The ability to combat AMR microbial communities is, however, evolving with the use of multi-omics, a new generation of interventions, such as antimicrobial peptides and nanoparticles, natural products, and phage-based therapies. Instead of only curing infections, scientists are starting to desaturate the biofilm communities that support them pre-emptively, which is an important step toward the future generation of solutions for persistent pathogens.
